# Training Veterans to Provide Peer Support in a Weight-Management Program: MOVE!

**DOI:** 10.5888/pcd10.130084

**Published:** 2013-11-07

**Authors:** Marlyn Allicock, Lindsey Haynes-Maslow, Carol Carr, Melinda Orr, Leila C. Kahwati, Bryan J. Weiner, Linda Kinsinger

**Affiliations:** Author Affiliations: Lindsey-Haynes-Maslow, Bryan J. Weiner, The University of Texas School of Public Health, Division of Health Promotion and Behavioral Sciences; Carol Carr, University of North Carolina at Chapel Hill, Lineberger Comprehensive Cancer Center; Melinda Orr, Linda Kinsinger, Veterans Health Administration, Durham, North Carolina; Leila C. Kahwati, Veterans Health Administration, Durham, North Carolina; and RTI, International, Research Triangle Park, North Carolina.

## Abstract

**Introduction:**

The Veterans Health Administration (VHA) has implemented MOVE!, a weight-management program for veterans designed to address the increasing proportion of overweight and obese veterans. The objective of our study was to determine whether peer support employing motivational interviewing (MI) could positively influence lifestyle changes, thus expanding the reach of the MOVE! program. We describe the initial evaluation of the peer training program.

**Methods:**

We developed an MI peer counselor training program for volunteer veterans, the “Buddies” program, to provide one-on-one telephone support for veterans enrolled in MOVE!. Buddies were recruited at 5 VHA sites and trained to provide peer support for the 6-month MOVE! intervention. We used a DVD to teach MI skills and followed with 2 to 3 booster sessions. We observed training, conducted pre- and posttraining surveys, and debriefed focus groups to assess training feasibility.

**Results:**

Fifty-six Buddies were trained. Results indicate positive receipt of the program (89% reported learning about peer counseling and 87% reported learning communication skills). Buddies showed a small improvement in MI self-efficacy on posttraining surveys. We also identified key challenges to learning MI and training implementation.

**Conclusions:**

MI training is feasible to implement and acceptable to volunteer Buddies. Trainers must assess how effectively volunteers learn MI skills in order to enhance its effective use in health promotion.

## Introduction

The worldwide prevalence of overweight and obesity has increased to epidemic levels (1). Approximately 70% of US veterans, a percentage similar to that of the nonveteran population, are overweight or obese defined as a body mass index of 25 kg/m^2^ or more (2). Recent Veterans Health Administration (VHA) data from fiscal years 2010 and 2011 suggest that the prevalence of overweight and obesity is over 80% among veterans receiving VHA care (L. Kahwait, written communication, October 2012), making health promotion, including weight management, a priority for the VHA.

The MOVE! Weight Management Program for veterans, initiated in 2006, is an evidence-based clinical obesity treatment program delivered by a multidisciplinary team that takes a comprehensive approach to weight loss and maintenance by promoting behavior change, healthful nutrition, physical activity, and psychological well-being. Since the VHA’s dissemination of MOVE! in 2006, modest positive effects on weight change at 6 months have been reported, for example, in 2009 for 98% of the 155 VHA medical centers providing MOVE! (3,4). A recent study looked at weight changes resulting from the program (5). Researchers have also examined participant retention (6), reach and effectiveness (7), and variations in program implementation (8–10).

Other studies have sought to enhance MOVE! components and outcomes (11,12). One pilot study used a quasi-experimental design with 195 veterans at 2 VA clinics for 6 months to demonstrate a greater effect for fruit and vegetable intake for participants receiving an enhanced program compared with the usual MOVE! program (12). The control group received the standard MOVE!, and the intervention group received MOVE! plus 4 tailored newsletters and motivational interviewing (MI) calls from clinic staff. The intervention group reported a significant increase in servings of fruits and vegetables compared with the control group (1.7 vs 1.2, *P* ≤ 0.05). However, process evaluation revealed that clinical staff lacked adequate time to conduct the calls: fewer than half of enrolled participants had been called. Moreover, anecdotal evidence suggested that the necessity of conducting calls with MOVE! participants may have been inhibiting clinical staff’s motivation to enroll more patients in MOVE!.

Several studies have tested methods to enhance peer support for veterans for diabetes self-management and chronic posttraumatic stress disorder (PTSD) (13,14) which suggests a role for veterans in providing peer support for behavior change in outpatient settings. Additionally, peer support has been shown as a key factor in behavior change (15,16). Given these findings, we created a peer support intervention that used a motivational interviewing approach and was delivered by veteran volunteers to address barriers to implementation among clinic staff in the MOVE! program.

Motivational interviewing (MI), originally developed for counseling addictive behaviors, is now being adapted for chronic disease management and health promotion (17,18). MI helps recipients address ambivalence about behavior change, barriers to change, and potential sources of motivational resources (17,19). The counselor establishes a safe, nonconfrontational, supportive climate through reflective listening rather than through persuasion or advice to allow the patient to weigh pros and cons of change. MI supports weight management behaviors for adult weight loss (19,20), healthful eating (21), childhood obesity prevention, and weight management (22,23). A limitation of typical professional-led MI interventions is the high cost and limited availability of trained counselors. Training lay volunteers and peers is a potentially cost-effective complement to existing behavior-change programs and has the capacity to give programs a broader reach than programs that rely solely on health professionals. Peers can be effective through their understanding of the targeted group’s needs and concerns and by offering valuable social, emotional, and tangible support (24).

Researchers at the University of North Carolina at Chapel Hill, in partnership with the VHA National Center for Health Promotion and Disease Prevention, developed and tested the dissemination of an MI-based peer counselor program to provide support to MOVE! patients in a 5-year dissemination study. This article describes the MI peer counselor program, MOVE! VETS (volunteer education and tailored self-management and support), and provides an initial evaluation of this component of the MOVE! program. The objective of our study was to determine whether peer support employing MI could positively influence lifestyle changes thus expanding the reach of the MOVE! program.

## Methods

MOVE! VETS was a group-randomized trial conducted at 10 VHA facilities that compared 2 different models for disseminating and implementing MOVE!*.* One study arm provided the standard MOVE! program and the other provided MOVE! plus 4 tailored newsletters and MI-based peer counseling, which was provided in person or by telephone by veteran volunteers. Randomization was by VHA facility to limit exposure to the intervention among control participants and to maximize efficiency in peer counseling training and in assessing organizational level implementation barriers and facilitators.

We recruited 10 VHA sites and formed 5 pairs matched by size, complexity, and geographic region. In each pair, 1 site was randomized to standard MOVE! and 1 to receive MOVE! VETS. Eligibility of veteran volunteer peer counselors (MOVE! Buddies) at the 5 intervention sites was based on successful participation in MOVE! and receiving services at VHA facilities (therefore no longer on active duty). Veterans who have completed the MOVE! program and achieved weight loss were nominated to be Buddies by the MOVE! facility coordinator or the facility site investigator, or they volunteered. Buddies completed standard VHA background screening for appointment as “without compensation employees” and completed the MI training before being assigned to work with MOVE! participants. No monetary incentives were provided. Each Buddy was expected to have up to 5 MOVE! participants, and each facility recruited 10 to 15 Buddies for the 6-month intervention. Facility coordinators, MOVE! staff, or research study staff were responsible for program implementation. This study was approved by all participating VHA facility institutional review boards and by review boards at the national coordinating centers (Durham Veterans Affairs Medical Center and the University of North Carolina at Chapel Hill).

The peer counselor training program was adapted from a similar program developed for the National Cancer Institute’s Body & Soul program (25), which trains peers in African American churches to support healthful diet changes for fellow church members. Four focus groups were conducted in 2 rounds with veterans from 2 geographically distinct regions. In the first round, participants from 2 focus groups (n = 9) focused on issues related to content of the DVD program, choice of narrator and characters, and incorporation of the VHA context into the program. The same 9 focus group participants reviewed the developed DVD to provide suggestions for revision. Questions for round 2 focused on participant comprehension of the DVD segments, reactions to characters and vignettes (eg, liking, credibility, similarity, relevance), and acceptability for veterans.

A coordinator’s guide for MOVE! program staff provided program logistics, training instructions, supplemental activities, and resources for training. A Buddy manual served as a written companion to the DVD and covered topics such as confidentiality, Buddy role, explanation of skills, and available MOVE! program resources. The DVD and manual focused on a set of core MI communication skills in the context of the MOVE! objectives promoting healthful eating and physical activity.

Central coordinating center research staff provided technical assistance to facility coordinators regarding content and logistics prior to the initial training and as needed for follow-up sessions. Four of the 5 coordinators had had previous training in MI techniques. The initial trainings were conducted from January 2009 through April 2010. The training was managed by the facility coordinators and provided as either a 1-day intensive training or in 2 parts (8 hours total) held at VHA facilities. Two to three 1-hour additional booster trainings were conducted during the intervention period to reinforce skills, provide additional information, assess competence, and troubleshoot problems. Training content varied by site based on the needs of Buddies.

The core training included 1) a general description of the MI approach and its spirit of being nonjudgmental and allowing the participant to express the argument for change; 2) open-ended questions; 3) reflective listening; 4) feedback from instructor to participant about performance and learning skills and to ensure that instructors elicit information from participants to assess content delivered and clarity of skills being learned; 5) tools for building motivation and promoting “change talk”; 6) discussion of barriers and menus of options, rather than single solutions, for addressing barriers; and 7) plans for follow-up. Each module included instruction in each skill, role-playing vignettes illustrating each skill in the context of an MI call, and interactive exercises designed to facilitate skill practice and confidence building. Buddies received a copy of the DVD and manual that reviewed the skills, included the interactive exercises, and provided a protocol (Appendix) for the conversations with MOVE! participants.

### Measures

Demographic information about the Buddies included age, sex, race/ethnicity, years of military service, branch of service, and current occupation. A brief pretraining survey rated Buddies’ confidence about their role as peer counselors (0 = not at all confident, 10 = very confident); and open-ended questions queried reason for volunteering and knowledge of peer counseling. At posttest, questions included Buddies’ confidence about their role (0 = not at all confident, 10 = very confident); extent to which the training helped in their role as peer counselors (0 = not at all, 10 = very much); perceptions of whether they learned valuable skills (“strongly agree” to “strongly disagree”); and open-ended questions about specific skills learned and skills that needed additional work.

Immediately following the training, a research team member conducted debriefing sessions with the Buddies. These sessions lasted approximately 30 minutes and used an interview guide to provide consistency in the questioning but with latitude to probe for detail. Questions were designed to capture participants’ reaction to the DVD training as a tool for teaching peer counseling skills and preparing them for their role and for raising any program concerns. A research team member observed the trainings conducted by facility coordinators at each site, document protocol adherence, duration of training sessions, participant responsiveness, and other training issues. After Buddies began contacting participants, a research team member conducted semistructured interviews with all 5 intervention site coordinators. Questions concerned use of the training materials, facility readiness to implement the trainings, and barriers and facilitators to program implementation.

Qualitative data (telephone interviews and training debriefings) were audiotaped and transcribed. Two team members summarized responses and conducted analyses to identify recurring themes. Categorical variables (from demographic survey, pre-and posttraining surveys, and training observation) were summarized by frequency and percentage.

## Results

A total of 56 veterans were trained as Buddies at the 5 intervention sites. Buddies ranged in age from 42 to 78 years (mean, 59 years) (Table) and were predominantly male (88%) and white (73%). Just under half had served in the Army (43%), and just over half were currently retired or semiretired from military service or other employment (52%). Length of military service varied from 1.5 months to 35 years (mean, 6.68 years; median, 5 years).

**Table:** Demographic Characteristics of Veterans Trained as MOVE! Buddies (N = 56), January 2009 – April 2010^a^

Characteristic^a^	n (%)
**Age**	42–78 (59.53)
**Sex**	
Male	49 (12.5)
Female	7 (87.5)
**Race**	
W	41 (73.2)
Black	8 (14.3)
Other	4 (7.1)
Unknown	3 (5.4)
**Service branch**	
Army	24 (42.9)
Air Force	15 (26.8)
Navy	14 (25.0)
Marine Corps	3 (5.4)
Duty status	
Retired/semi-retired	29 (51.8)
Employed/volunteer	19 (33.9)
Disabled/unemployed	5 (8.9)
Unknown	3 (5.4)
**Completed Leadership Development Course as part of military training?**	
Yes	25 (44.6)
No	24 (42.9)
Unknown	7 (12.5)

a Length of military service for participating veterans ranged from 1.5 months to 35 years (mean, 6.68 years).

Pre-training survey results showed that Buddies volunteered to help other veterans with weight management because someone asked or recommended them, but most volunteered because they wanted to help other veterans. Some Buddies felt there was a high need for the program and others wanted to help or improve themselves. They felt confident serving as peer counselors (mean rating, 7.9).

Posttraining survey results showed a small change in MI self-efficacy after training (posttest mean, 8.4). Buddies felt the training prepared them to be peer counselors; 89% enjoyed learning about peer counseling, and 87% felt they learned valuable communication techniques. Overall, most enjoyed learning about MI-based communication skills. One Buddy said the training helped him “learn good techniques to spend more time listening, less time talking.” The use of role-playing and practice scenarios was helpful in understanding MI concepts. Buddies liked the training DVD and thought the information was helpful and that they could relate to the actors in the DVD.

Challenges to the training were related to the DVD content and learning about MI. Some found the DVD scenarios too scripted and did not think the DVD actors adequately represented veterans in the program. One commented, “The DVD instructor is too thin – didn’t look like he’d ever had a weight problem so I couldn’t relate.” Some had trouble grasping MI concepts, specifically reflective listening. A few felt that reflective listening was “mundane” and sounded like they were “talking down” to the veterans. Buddies were encouraged not to simply “parrot” back what was being said or to use “it sounds like . . .” with every reflection. They were taught to focus on the emotion being communicated (eg, “you’re frustrated that you’re not losing weight faster”). Strategies like these allowed Buddies to sound ‘less clinical” or “preachy” and therefore more authentic. Two felt uncomfortable role-playing in front of other trainees. A few pointed out that the training should include guidance on how to deal with difficult situations they might encounter (eg, communicating with a veteran who has PTSD or depression). During the debriefing, Buddies made suggestions for additional training including opportunities to review practice exercise questions individually and then review in group, breaking into smaller groups for role-playing, and monthly refreshers to review material.

Three sites conducted 1-day trainings, and 2 sites divided trainings over 2 days. Attendance ranged from 4 to 11 (average, 7). Facility coordinators followed the training protocol closely (eg, discussed role of Buddies, confidentiality issues, reviewed all 4 MI communications skills, included practices).

We interviewed facility coordinators to get their reactions to training. Overall, the 5 facility coordinators thought the training materials were helpful, including the DVD and practice scenarios. One coordinator commented, “Given that the material was brand new to all of my peer counselors, I would say it was very easy to understand. . . . Probably 90% walked away with some of the basic concepts of motivational interviewing.” Additionally, most coordinators felt the DVD characters were appropriate: “I thought the narrator was very good. [Actors] came from different military backgrounds, which was nice, because every peer counselor could bond with that person.” All of the facility coordinators felt their facility was ready to implement the Buddy program at the conclusion of the training.

Although the majority of feedback about the training was positive, 1 suggestion was to include veterans with chronic illnesses or mental health or substance-abuse issues. Other challenges to the training included scheduling (3 facilities) and space and equipment availability (2 facilities). Learning about MI was difficult for some Buddies because they wanted to help by sharing their own experiences rather than practicing reflective listening:

A lot of these veterans came in wanting to help, and they had a tendency to —even though during the training we tried to make sure that they understood their role and responsibility was not to be a content expert or not to force anybody to do anything. But mainly be there to support their participants. Some of them really wanted to give a lot of advice and be prescriptive.

Several recommendations were made, including having more structured follow-up training sessions, distributing summary slides after each lesson in the DVD training, and having the DVD show more situations with “challenging” Buddies.

## Discussion

This training evaluation was designed to assess the value of a peer support program for veterans as part of MOVE!. Results suggest that the training program is feasible to implement and acceptable to veteran trainees. Other studies have employed veterans in a support role to assist other veterans for chronic disease management. Heisler and colleagues (13) tested whether peer support is a feasible and useful strategy for improving the self-management and health outcomes of patients with diabetes. Another study surveyed veterans treated for PTSD to examine the relationship between PTSD and social support (15). Findings indicated that veteran peers were an important and highly valued component of social networks for veterans with PTSD (14). Other studies have used MI with veterans for suicide prevention (26) and obesity prevention (27, but none to our knowledge have trained veteran peers to use MI.

In our study, Buddies reported their greatest challenge was difficulty learning reflective listening. Other research with nonveteran peers using MI reported this skill as challenging for first-time learners (28). As with all learning, additional time and training led to increased skill. We could not document this because it was only feasible to observe the initial training and not follow-up trainings. Another common issue (12) was the challenge of altering the expectations of first telling and sharing one’s own success stories (“if I can do it, so can they”) rather than understanding the needs, goals, and motivations of the veteran needing support. This issue was raised at each training site by Buddies who believed that telling their own success story was what would change the behaviors of other veterans. Buddies who had been helped by non-MI programs may have felt that MI techniques could work better if reinforced with military-type training: “You are told what to do and you do it.” It was therefore important that the training address the issue of simply telling what to do versus the focus of MI, which draws out a person’s needs, motivations, and barriers to change to address their own issues with weight management.

Finally, trainees said the training should address issues such as weight management in the context of PTSD and depression. Although the training addressed asking about barriers and stressors, trainees felt that new learners needed concrete examples of issues relevant to a veteran population. The follow-up trainings and oversight by the facility coordinators allowed for trouble-shooting the issues that arose when Buddies were matched with participants. Furthermore, Buddies were instructed to contact their MOVE! facility coordinator should participants present issues related to depression or PTSD or other medical issues so appropriate professional evaluation and intervention could be provided.

This study’s limitations included the inability to measure the fidelity of MI skills used in the MOVE! Buddy calls. Although MI skills may be initiated with a few days of training, ongoing learning support for maintenance and increased skill is required (29). In this study, coaching was done by facility coordinators to improve MI adherence and to help solve any problems Buddies identified. The research team only observed initial training at all sites to pinpoint common concerns to help each site with training. This article presents a description of the training component, but it does not include data on long-term performance and impact. The objective was, instead, to report on the feasibility of using MI as an approach for training veterans to provide support and to understand the barriers to providing that support and what facilitated delivery of support. Data regarding patient satisfaction with the program, exposure, and impact will be reported elsewhere.

Given the numerous health-related problems linked to obesity, it is important to understand whether and how peer support interventions can help promote behavior change for weight management. The MI training proved feasible to implement and was accepted by veteran trainees. The reach of MOVE! could be enhanced if the MI peer-support intervention is at least as effective as traditional MOVE!.

## Appendix. MOVE! Buddy: Road Map for Telephone Conversations

Use the roadmap as a guide to the main points of the MOVE! Buddy counseling conversation. Each conversation will take its own path, so you may not follow the roadmap exactly. For example, you might talk about values, importance, and confidence in a different order.

Introduction

Introduce yourself as a MOVE! Buddy*.*

Explain purpose of call: Say that you understand the veteran has signed up to talk with a MOVE! Buddy*.*

Ask if they still want to talk. If so, set a time and place for the meeting or call.

Talk about healthy eating and exercise.

Ask why the veteran signed up for MOVE! *Buddy* counseling.

Listen and reflect.

Ask about what kinds of foods the person eats every day.

Ask about what kinds of exercise they do.

Ask them what they like (or dislike) about fruits, vegetables, and low-fat foods.

Ask if the person knows the amount of fruits and vegetables he/she should eat. If not, ask if they would like to know the recommendation.

Ask if the person knows the amount of exercise he/she should do. If not, ask if they would like to know the recommendation.

Listen and reflect.

Talk about values.

Explain that making a lifestyle change is easier if the change is tied to one’s values.

Ask what the veteran’s top three values are and what they mean to the person.

Listen and reflect.

Ask what connection, if any, the person sees between these values and eating more healthfully and getting more exercise.

Reflect on the connection or lack of connection.

Rate importance and confidence.

Ask the veteran to rate the importance of eating more fruits and vegetables on a scale of 0 to 10.

Ask why the person chose ________ and not a lower number, like a 1 or a 2.

Listen and reflect.

Ask what it would take to move this number a little higher.

Listen and reflect.

Ask similar questions about confidence. Listen and reflect.

Rate importance and confidence.

Ask the Veteran to rate the importance of eating more fruits and vegetables on a scale of 0 to 10.

Ask why the person chose ________ and not a lower number, like a 1 or a 2.

Listen and reflect.

Ask what it would take to move this number a little higher.

Listen and reflect.

Ask similar questions about confidence. Listen and reflect.

Create an action plan/summarize/close.

Ask if the person has some ideas for ways to eat more healthfully and get more exercise.

Listen and reflect.

If not, *with permission*, suggest some ideas and ways to get more information.

Ask if any of these ideas might work for them.

Listen and reflect.

Summarize the key parts of the conversation.

Ask if it would be okay to call the person in about two weeks.

End the conversation.
